# Blue-Green Algae (*Spirulina platensis*) Alleviates the Negative Impact of Heat Stress on Broiler Production Performance and Redox Status

**DOI:** 10.3390/ani11051243

**Published:** 2021-04-26

**Authors:** Eman S. Moustafa, Walaa F. Alsanie, Ahmed Gaber, Nancy N. Kamel, Abdulaziz A. Alaqil, Ahmed O. Abbas

**Affiliations:** 1 Department of Animal Production, Faculty of Agriculture, Cairo University, Giza 12613, Egypt; aabbas@kfu.edu.sa; 2 Department of Clinical Laboratories Sciences, College of Applied Medical Sciences, Taif University, P.O. Box 11099, Taif 21944, Saudi Arabia; w.alsanie@tu.edu.sa; 3 Department of Biology, College of Science, Taif University, P.O. Box 11099, Taif 21944, Saudi Arabia; a.gaber@tu.edu.sa; 4 Department of Animal Production, National Research Center, El Buhouth St., Dokki, Giza, Cairo P.O. Box 12622, Egypt; nancy.nk3@gmail.com; 5 Department of Animal and Fish Production, College of Agricultural and Food Sciences, King Faisal University, P.O. Box 420, Al-Ahsa 31982, Saudi Arabia; aalaqil@kfu.edu.sa

**Keywords:** cyclic heat stress, *Spirulina platensis*, redox status, serum metabolites, blood hematology, meat quality, broiler

## Abstract

**Simple Summary:**

Heat stress is the leading cause of poor broiler productivity in tropical and subtropical countries. To face such stress, natural antioxidant feed additives are attracting interest due to their high effectiveness and safety. Dietary algae *Spirulina platensis* have received much attention in the last decade due to its high protein content. The effectiveness of (*Spirulina platensis*) as a feed additive to alleviate the negative impacts of heat stress on production performance was investigated. Under heat stress conditions, *Spirulina* supplementation improved broiler productivity and was able to bring back redox balance. It can be inferred that *Spirulina* can be used as a natural antioxidant supplementation to heat-stressed broilers for improving the production performance and modulating serum metabolites to bring them to the normal values.

**Abstract:**

The modern broiler industry faces huge challenges to keep high production quality and quantity, especially under environmental heat stress conditions. The negative effect of heat stress on broiler productivity is mediated by oxidative stress induction. The blue-green alga (*Spirulina platensis*) has many applications in poultry nutrition with the high levels of bioactive antioxidant compounds, which can alleviate the oxidative stress damage induced by high ambient temperature. The current study was designed to investigate the effects of dietary *Spirulina* inclusion at different levels on growth performance, redox status, carcass traits, meat quality, blood hematology, and metabolites profile of broilers subjected to cyclic heat stress. A total of 300 one-day-old Cobb-500 broiler chicks were recruited. Starting from day 21 to 42 of age, birds were randomly divided into five treatment groups with 6 replicates × 10 birds per group, where the first one was provided with the basal diet and reared under normal thermal conditions (23 ± 1 °C) to serve as a negative control. Meanwhile, the other four groups were exposed to cyclic heat stress (34 ± 1 °C for 8 h per day) and were fed a basal diet supplemented with *Spirulina* at a concentration of 0, 0.5, 1 or 1.5%. *Spirulina* supplementation to heat-stressed broilers was able to alleviate the negative impacts of heat stress on the final average daily gain, body weight and feed conversion ratio, with the best impact observed among the chickens fed 1% *Spirulina*. Hematological results indicate increasing hemoglobin and hematocrit levels with *Spirulina* supplementation compared to the non-supplemented stressed group. Further, *Spirulina* supplementation significantly influenced blood lipid metabolites marked by reduced serum cholesterol and low-density lipoprotein (LDL), and increased high-density lipoprotein (HDL) levels. The lipid peroxidation level was reduced (*p* < 0.05), while the antioxidant enzyme activity was increased with *Spirulina* supplementation to the heat-stressed group. *Spirulina* supplementation at 0.5 or 1% improved carcass dressing, breast and leg percentages. It can be concluded that dietary *Spirulina* supplementation at 0.5 or 1% to broiler reared under heat stress conditions can effectively improve broiler production performance and balance the redox status.

## 1. Introduction

One of the challenges facing the modern broiler chicken industry is the high ambient temperatures, especially in regions depending on the open production system [1]. Modern, rapidly growing broiler genotypes are more sensitive to heat stress due to higher metabolic activity, generating more body heat [2]. Lin et al. [3] reported that the optimal production temperature for growing broilers ranged from 18 to 22 °C, while heat stress may occur at an average of 30 °C. Heat stress reported having a negative influence on broiler chicken digestion, nutrient absorption, carcass characteristics, development of immune organs, immune response and survival [4,5,6,7,8]. Broilers exposed to acute heat stress at the market age have been reported to have lower growth performance accompanied by serum metabolites disorder and imbalance redox status [9]. Furthermore, meat quality was reported to be impaired in broilers subjected to chronic heat stress [10,11]. Decreased ultimate pH and increased lightness (L*), cooking loss and shear force are the commonly reported negative effects of heat stress on broiler meat quality [11]. Eventually, heat stress threatens the profitability of poultry in many countries of the world, particularly during the summer months [12,13]. Therefore, there is a continuous endeavor for functional food materials that can be safely utilized to enhance the health and wellbeing of birds subjected to heat stress conditions.

Heat stress is known to break the body redox balance, resulting in the generation of reactive oxygen species (ROS), which induces oxidative damage, and subsequently affects nutrient absorption and metabolism negatively [14]. To overcome the detrimental effects of heat stress and the subsequent oxidative stress induction on poultry production, many intervention strategies have been suggested but with variable or inconsistent outcomes [5]. The fundamental ground and ultimate goal for choosing a mitigation strategy confronting heat stress are to reduce oxidative stress and relieve its tissue-damage impact. Numerous additives have been proposed to improve the performance of birds suffering from heat stress. Mineral and vitamins [15,16], organic acids [17,18], phytogenics [19,20], probiotics [21] and prebiotics [22] are some of the presented additives used to mitigate the negative impact of heat stress on poultry performance. Furthermore, phytochemicals with antioxidant activity, such as polyphenols, were presented as a potentially effective feed additive to confront heat stress in poultry [14].

*Spirulina platensis* is a blue-green alga rich in protein content, vitamins, minerals and phytopigments [23]. It can be produced in marine or freshwater aquatic systems, with marginal land requirement [24], which reduces the conflict of using limited farmland in cultivating animal feed. *Spirulina* is currently presented as an effective alternative dietary source to substitute the costly supplies of fishmeal and fish oil required in the poultry diet [25,26]. Tavernari et al. [27] demonstrated the potentials of using *Spirulina* as an alternative dietary ingredient for formulating diets that require higher amounts of essential amino acid and metabolizable energy. It can replace up to 15% of broiler diets as a partial substitute of traditional protein sources without negatively affecting production performance or meat quality [28,29]. Moreover, *Spirulina* contains bioactive compounds (e.g., gamma-linoleic acid, phycocyanins, phenolic acids, β-carotene and chlorophyll) [30]. Agustini et al. [31] found that dried *Spirulina* contains high bioactive compounds (i.e., phenolic, flavonoid, saponin, triterpenoid and steroid), which contribute to its high antioxidant activity. Furthermore, Park et al. [32] reported that applying the *Spirulina* addition to broiler chicken diets could enhance growth efficiency, nutrients digestibility, increasing antioxidant enzyme activity, modulating cecal microflora and reducing excreta noxious gas emission. Dietary supplementation of *Spirulina* to chickens reared under chronic heat stress conditions may mitigate the adverse effects of such stress on the growth performance and the immune status of both local chicken strains [33] as well as commercial strains [34,35]. Hence, the present study was designed to investigate the effect of dietary inclusion of *Spirulina platensis* to heat-stressed broiler diets on growth performance, carcass traits, meat quality, hematological and serum biochemical profile.

## 2. Materials and Methods

### 2.1. Experimental Material

The *Spirulina platensis* microalgae were obtained from a commercial supplier (Inner Mongolia Rejuve Biotech. Co., Ltd., Ordos, China) in the form of freeze-dried powder. According to the manufacturer, the nutrients composition of *Spirulina* powder was 5.6, 56.4, 7.2, 0.02 and 7.5% for moisture, protein, ether extract, fiber and ash, respectively.

### 2.2. Experimental Design

The ethical committee of King Faisal University, Al-Ahsa, Saudi Arabia, approved the experimental procedures. It is committed to favoring animal rights and reducing the discomfort, pain, and misery of the birds. A total of 300 unsexed one-day-old Cobb-500 broiler chicks with an average body weight of 45 ± 1.3 g were obtained from Al Watania Poultry Co., kept under normal controlled conditions and nourished with corn–soybean meal basal diet ad libitum with continuous access to fresh water. The basal diet was formulated to meet the nutritional requirements according to the NRC [36] and the management guide of Cobb-500 broiler and offered in a mash form. Starting from the 22nd day and until the 42nd day of the breeding, the chicks were randomly distributed (considering the equal ratio of males to females) into five treatments with 6 replicates (10 chickens each). Each replicate of birds was reared in a floor pen (0.90 lengths × 0.90 widths × 0.38 m height) and relative humidity of 50 ± 5%. Birds were fed ad libitum. In a temperature-controlled room, the broilers in the thermoneutral group (TN) were raised at 23 ± 1 °C and 50 ± 5% relative humidity receiving a corn–soybean meal basal diet (Table 1). In another temperature-controlled room, the heat-stressed groups (HS) broilers were exposed to cyclic heat stress at 34 ± 1 °C for 8 h per day (from 9:00 a.m. to 5:00 p.m.) and fed the basal diet mixed with either 0, 0.5, 1, or 1.5% of *Spirulina platensis* (SP) powder, respectively. After the high heatwave, the excess heat was removed by increasing ventilation and evaporation cooling after the heat challenge. Birds were kept on an illumination program of 23 L: 1D.

### 2.3. Production Performance Parameters

Feed consumption was recorded daily, and the birds were weighed by replication after fasting for 12 h. Feed conversion ratio and average daily gain were calculated at the end of the experiment. The mortality rate of birds was recorded in all the treatments, and the European broiler index (EBI) was estimated following Islam et al. [37] formula; European broiler index (EBI) = daily gain × survival rate/10 × feed conversion ratio.

### 2.4. Blood Hematological, Biochemical and Redox Profile Analysis

At the end of the trial, 12 blood samples per treatment (2 birds per replicate) were obtained from the brachial vein into heparinized and non-heparinized tubes. The serum was separated by centrifugation at 1500× *g* for 10 min at 4 °C and stored at −18 °C until further analysis. Serum cholesterol, triglyceride, high-density lipoprotein (HDL), creatinine and urea were determined using commercial diagnostic kits according to the manufacturer guidelines (Diamond Diagnostics Company, Cairo, Egypt). Low-density lipoprotein (LDL) was determined according to the formula of Friedewald et al. [38] (LDL = total cholesterol (TC) minus high-density lipoprotein (HDL) minus triglycerides (TGs)/5 in (mg/dL)). The activities (U/L) of alanine aminotransferase (ALT) and aspartate aminotransferase (AST) enzymes were measured using commercial kits (BioDiagnostic, Giza, Egypt) according to the method described by Reitman and Frankel [39]. Serum glutathione reduced concentration (GSH), superoxide dismutase (SOD) activity and total antioxidant capacity (TAC) were measured using commercial kits (Nanjing Jianheng Bioengineering Institute, Nanjing, Jiangsu, China). The serum malondialdehyde (MDA) concentration was determined following the thiobarbituric acid reaction method using a commercial colorimetric assay kit (Nanjing Jiancheng Bioengineering Institute, Nanjing, Jiangsu, China).

Total red blood cells (RBCs) were counted by a Bright-Line™ hemocytometer (American Optical, Buffalo, NY, USA), using a light microscope at 1900× magnification. Before counting, blood samples were diluted 200 times with physiological saline. The hemoglobin concentration was calculated using the cyanomethemoglobin method [40]. For the hematocrit calculation, Wintrobe hematocrit tubes were used, blood samples were centrifuged at 1900× *g* for 20 min and 4 °C, then the hematocrit values were determined on the graduated scale by reading the packed cell volume. The values of MCV, MCH and MCHC% were calculated using the following formulas: The average volume (size) of RBC (MCV, mm^3^) = (hematocrit%/RBC] × 10. The average weight of hemoglobin in RBC (MCH, pg) = (hemoglobin concentration (g/dL)/RBC) × 10. The average concentration of hemoglobin in the RBC (MCHC, %) = (hemoglobin (g/dL)/ hematocrit%) × 100.

### 2.5. Carcass Characteristics

Twelve chicks were taken randomly from each treatment at the end of the trial, two birds per replicate, weighed individually and slaughtered. At 54 °C for 2 min, the chicks were scalded and then de-feathered, and eventually, their heads were cut. Breast muscles and abdominal fat were immediately separated from the hot carcass. The internal organs from the beginning of the esophagus to the end of the outlet were separated in a detailed anatomical fashion [41]. Using a sensitive balance of 0.1 g, the intestines were cleaned and weighed, and the data were expressed as a percentage of carcass weight. The dressing percentage was calculated by dividing the clean carcass weight by the living body weight. The breast muscle, thigh muscle, abdominal fat, gizzard, liver, heart and small intestine weights were estimated as a percentage of carcass weight.

### 2.6. Meat Quality Measurements

The meat color of breast muscle samples at 45 min postmortem were determined using a color reader (Minolta CR-10, Konica Minolta, Tokyo, Japan) to measure the meat color applying the CIELAB method (L* = lightness; a* = redness; b * = yellowness). At a depth of 1 cm, a pH meter (HI9125, HANNA Instruments, Venice, Italy) was used to assess the pH value at 24 h postmortem. Cooking loss and shear strength of samples were assessed at 24 h postmortem as previously described by Lu et al. [42]. Briefly, after slaughter, muscle samples were weighed, hung in a sealed plastic bag at 4 °C for 24 h and weighed again. The drip loss was expressed as the percentage of weight loss during storage. The samples were eventually put into a lined plastic bag and heated for 20 min in a water bath to reach an interior temperature of 75 °C. The samples were weighed after cooling to room temperature, and cooking loss was expressed as the percentage of loss of weight after cooking. A C-LM3 texture analyzer (Northeast Agricultural University, Harbin, China) was then used to assess shear intensity.

### 2.7. Statistical Analyses

Data for all variables excluding the normal temperature treatment (thermoneutral) were analyzed using one-way analysis of variance (ANOVA) using the general linear model (GLM) procedure of SAS/STAT^®^ 9.3 software (Copyright© 2021, SAS Institute Inc., Cary, NC, USA). A single degree of linear freedom contrast was used to evaluate the effect of thermoneutral versus cyclic heat stress for birds on the basal diet without *Spirulina*. The results obtained were expressed as mean of standard error (SEM), and significant differences between treatment means were determined using Tukey’s test. Each floor pen was considered as an experimental unit for growth performance measurements. Meanwhile, for other measurements, each bird was considered as an experimental unit. Significant values were determined at *p* < 0.05.

## 3. Results

### 3.1. Growth Performance

The effects of the different dietary supplementation levels of *Spirulina platensis* on the growth performance of Cobb-500 broiler chickens subjected to cyclic heat stress are presented in Table 2. Growth performance was negatively influenced by heat stress exposure. Feed intake was significantly reduced (*p* < 0.037) by 12% in the HS group compared to the thermoneutral group with no effect of *Spirulina* supplementation. The average daily gain and final body weight were significantly reduced (*p* < 0.009 and *p* = 0.012, respectively) in HSgroup compared to the thermoneutral. Under heat stress conditions, dietary *Spirulina* supplementation at the level of 1% significantly increased both average daily gain and final body weight by 27 and 19%, respectively, compared to the HS group. The other *Spirulina* supplemented groups, HS 0.5 and HS 1.5%, showed an increase (*p* = 0.031) in the average daily gain by 13 and 14% and higher (*p* = 0.034) final body weight by 16 and 15%, respectively, compared to HS group. Moreover, the feed conversion ratio was improved (*p* = 0.023) with *Spirulina* supplementation at different levels than HSgroup. European broiler index (EBI) is a function of daily gain, survival rate and feed conversion ratio. EBI was significantly reduced by heat stress exposure. Nevertheless, *Spirulina* supplementation significantly elevates EBI values with the best production efficiency achieved with 1% and 1.5%, followed by 0.5% supplementation level.

### 3.2. Hematological Parameters

The hematological profile of broiler chickens exposed to cyclic heat stress and supplemented with different levels of *Spirulina* is illustrated in Table 3. Chronic heat stress reduced (*p* = 0.047) the total number of broiler red blood cells (RBC), moreover, it showed significant negative effect (*p* = 0.041) on hemoglobin (HGB) and hematocrit (HCT) values with subsequent reduction (*p* = 0.019) of mean cell volume (MCV) and low (*p* = 0.033) mean cell hemoglobin (MCH) compared to the thermoneutral group. *Spirulina* supplementation to heat-stressed chickens, irrespective of the addition level, was able to improve) RBC, HGB and HCT values compared to the HS group. Meanwhile, MCV and MCH showed significant elevation in HS 0.5 and 1% *Spirulina* supplementation compared to the non-supplemented HS group.

### 3.3. Serum Biochemical Parameters

Broiler blood serum biochemical parameters of the different experimental groups are shown in Table 4. The results demonstrated the presence of significant changes in blood metabolites related to heat stress exposure. Results related to lipid metabolism showed significant increase in serum cholesterol (*p* = 0.015), LDL (*p* = 0.011) and triglycerides (*p* = 0.023) levels and a decrease in HDL (*p* = 0.003) level in HS group compared to the thermoneutral group. Serum concentrations of creatinine and urea, as well as the activity of AST, were significantly elevated in the HS group compared to the thermoneutral group. Results demonstrated that *Spirulina* supplementation generally revoked the negative impact of heat stress on serum lipid metabolites, liver function and kidney function. *Spirulina* supplementation was able to significantly decrease serum cholesterol, LDL, triglycerides, creatinine, urea concentrations and AST activity while increase HDL level compared to the non-supplemented HS group.

### 3.4. Blood Redox Status

The redox status of broiler chicken reared under cyclic heat stress with different levels of *Spirulina* supplementation is presented in Table 5. Heat stress disturbed the redox status of broiler chickens. The lipid peroxidation increased significantly (*p* = 0.021) with heat stress exposure, as indicated by the high levels of MDA. The activity of serum antioxidant SOD enzyme and GSH concentration were significantly reduced in the HS group by 1.5 and 1.6-fold compared to the thermoneutral group. Consequently, the total antioxidant capacity showed a 33% reduction (*p* = 0.040) in the cyclic-heat-stress-exposed group with 0% *Spirulina* supplementation compared to the thermoneutral group. Moreover, *Spirulina* supplementation, at all experimented levels, modulated the negative impact of heat stress on broiler redox status by reducing MDA concentration while increasing SOD and TAC activities and GSH concentration.

### 3.5. Carcass Characteristics

Carcass characteristics were calculated and presented in (Table 6). Dressing percentage, as well as breast and leg percentages, decreased significantly with heat stress exposure. No effect was found on the liver, heart and gizzard proportions among the different experimental groups. Meanwhile, carcass fat percentage was significantly lower (*p* = 0.11) in the HS group compared to the thermoneutral group. Under heat stress, dietary *Spirulina* supplementations at 1% improved carcass dressing percentage (*p* = 0.020) and breast (*p* = 0.032) proportion. Furthermore, the intestine percentage was higher (*p* = 0.05) by about 18% in *Spirulina* supplemented groups compared to the non-supplemented HS group. These findings indicate that *Spirulina* supplementation has a positive effect on carcass characteristics in broilers reared under heat stress, with the best outcome observed among chickens fed at 0.5 and 1% supplemented levels.

### 3.6. Meat Quality Measurements

Meat is the end product of the broiler industry. Thus its quality is crucial for the breeders’ final profitability. In the present study, meat quality parameters showed no changes across the HS group compared to the thermoneutral group (Table 7). Nevertheless, *Spirulina* supplementation, at all levels, increased (*p* = 0.31) the meat yellowness (b*) compared to the HS group.

## 4. Discussion

The results of the current study demonstrated the presence of significant negative effects of heat stress on broiler growth performance. Akbarian et al. [43] reviewed the side effects of heat stress exposure on poultry to be numerous and significantly influence both wellbeing and productivity of the birds. Reduction of voluntary feed intake, decreased energy availability, alteration of various nutrients digestibility and metabolism, as well as the disintegration of intestinal epithelium structure and function, are some of the direct negative impacts of heat stress exposure on poultry behavior and physiology [42]. Low feed intake and body weight gain, as well as increased feed conversion ratio and mortality, were reported in chickens subjected to cyclic heat stress from day 22 to 35 of age [10]. The negative effects of heat stress on broiler growth performance were suggested to be mediated by changes in the intestinal morphology and permeability rather than the alteration in feed intake [44,45]. Liu et al. [46] reported that chronic cyclic heat stress-induced intestinal damage and altered cecal microflora profile via oxidative stress induction. Acute heat stress was also reported to cause serious damages in chickens’ small intestine and liver [47]. Adding *Spirulina* to heat-stressed chickens significantly improved growth performance [35]. Although *Spirulina* supplementation in the current study did not increase feed intake, the supplementation improved feed conversion and subsequently the final bodyweight of the heat-stressed chickens. Hajati and Zaghari [48] reported a significant increase in the final body weight and the European production efficiency factor of Japanese quails supplemented with 5 g/kg *Spirulina*. A significant increase in growth rate and improvement in feed conversion ratio was reported in broiler-fed-*Spirulina*-enclosed diet with 5 or 10 g/kg feed [49]. Moreover, *Spirulina* supplementation at 0.25, 0.5, 0.75, or 1.0% linearly improved body weight gain, feed conversion ratio and European production index [32]. The positive impact of *Spirulina* supplementation on broiler performance can partially be justified by the high apparent metabolizable energy and amino acid digestibility of *Spirulina* supplemented diet [27], especially with the negative impact of heat stress on intestinal morphology and feed consumption. Moreover, *Spirulina* addition may beneficially alter intestinal microbial population with a reported increase in *Lactobacillus* sp. and a decrease in *E. coli* population [49], as well as improving gut morphology with higher villi length and increasing goblet cells [50]. The significant increase in relative intestinal weight observed in the present study with *Spirulina* addition could be involved in the adaptation to low feed intake, which subsequently plays a role in compensating the reduction in feed efficiency of heat-stressed birds [51].

Blood biochemical profiles can reflect different physiological changes in birds (e.g., species, age, season, nutrition, and physiological condition) [52]. In the present study, heat stress exposure elevates broiler serum cholesterol, LDL and triglycerides levels while reducing HDL levels. These results can be partially justified by activating the hypothalamic–pituitary–adrenal axis and the release of glucocorticoids, which mediate such serum biochemical changes to confront the excessive heat load [11]. Moreover, heat stress mediates multiple pathways involving glucose, amino acid and lipid metabolism [53] and is reported to increase liver triglyceride synthesis [54] and serum triglyceride and cholesterol levels [55]. Guo et al. [56] reported alteration of serum lipid metabolism in heat-stressed-indigenous slow-growing chickens. Plasma triglyceride and liver enzyme concentrations increased significantly in broilers exposed to chronic heat stress for 14 days [57]. *Spirulina* supplementation at 0.5 to 2% reported decreasing serum cholesterol, LDL, total lipids and triglycerides of broilers subjected to heat stress [34,35]. Moreover, adding *Spirulina* to broiler diet at 5 or 10 g/kg feed was reported to increase hemoglobin level with no effect on total RBC count [49]. The low level of hemoglobin observed in heat-stressed chicken can be justified by the negative effect of heat exposure on ion absorption (i.e., iron), leading to a reduction in hemoglobin formation [58]. Further, the rich minerals content [59] as well as the modulation of intestinal integrity and permeability [60] that *Spirulina* supplementation presents directly modified the blood hemoglobin level. From these findings, it can be concluded that *Spirulina* supplementation to heat-stressed broilers helps recover the normal blood metabolite and hematological profile.

The negative impacts of heat stress on bird physiology and behavior are generally mediated by the induction of oxidative stress and redox imbalance [5,43,46]. The current results demonstrated that chronic heat stress negatively disturbed the redox balance with increasing MDA levels and decreasing GSH concentration, the activity of SOD and the total antioxidant capacity. However, *Spirulina* supplementation, due to its bioactive antioxidant compounds [31], was able to bring back redox balance. Dietary *Spirulina* at 0.25, 0.5, 0.75, or 1.0% showed linear increase of broiler serum antioxidant enzymes (SOD and glutathione peroxidase) [32]. Furthermore, under chronic heat stress conditions, *Spirulina* supplementation to broiler chicken significantly elevates SOD and glutathione peroxidase while decreasing MDA levels [34,35]. It has been reported that *Spirulina* supplementation effectively activates antioxidant enzymes while reduces MDA production in rats subjected to oxidative stress induced by heavy metal [61]. They justified these effects to the bioactive compounds found in *Spirulina*, such as α-tocopherol, ascorbic acid, β-carotene and selenium [61].

Carcass composition and meat quality are largely affected by exposure to high environmental temperature conditions [10,11]. Lu et al. [62] investigated the effect of chronic heat stress on broiler breast muscle quality and reported a reduction in pH _45 min_, increased lightness, drip loss and intramuscular fat deposition. They further explained such changes by the negative impact of heat stress on the mitochondrial function that causes a decrease in fat and glucose aerobic metabolism and increases in glycolysis and fat deposition. Under our study condition, the lack of differences among groups in meat quality traits can be justified by birds adaptation to the cyclic heat stress imposed, especially under the relieving procedures that were practiced after the heat stress period (i.e., offering cool water and increasing ventilation to remove the excess heat). Chronic heat stress was reported to reduce the proportion of broiler breast muscle and increase the proportion of thigh muscle as well as increase fat deposition [11]. However, the present results showed that *Spirulina* supplementation to heat-stressed broilers at 1% improved carcass composition. Dressing percentage was improved by *Spirulina* addition at 2 g/kg feed with no effect on abdominal fat pad [50]. Hajati and Zaghari [48] supplemented *Spirulina* at the levels of 2.5 or 5 g/kg diet and reported increases in the relative weight of the breast in quail reared under normal environmental conditions. *Spirulina* supplementation increased the yellowness (b*) of meat, which can be attributed to its high carotenoid content [61,63]. Pestana et al. [64] reported higher yellowness values and total carotenoids in breast and thigh meats of chicken fed 15% *Spirulina*. The positive influence of *Spirulina* on carcass composition and meat quality can be justified by the improvement in energy partitioning in favor of muscle development. Furthermore, *Spirulina* has a positive impact on high nutrient content leading to improvement in feed efficiency and nutrient conversion to lean meat.

## 5. Conclusions

The present study demonstrated the negative impact of chronic cyclic heat stress on broiler growth performance, carcass composition and blood hematological and metabolite profiles. The negative impacts of heat stress on the studied broiler physiological aspects can be justified by the induction of oxidative stress. Dietary *Spirulina* supplementation to heat-stressed chicken positively affects different production performance aspects as well as hematological and biochemical parameters. Furthermore, *Spirulina* addition at 0.5 and 1% to broiler feed can be used to alleviate the negative impacts of heat stress on broiler carcass composition without any negative impact on meat quality. In addition, the blood biochemical and hematological profile of broiler exposed to chronic heat stress can be enhanced by *Spirulina* addition. Furthermore, *Spirulina* supplementation can balance the redox status of broilers reared under heat stress conditions and hence improve productivity.

## Figures and Tables

**Table 1:** Ingredients and nutrient composition used of the experimental diets as fed basis (days 22 to 42).

Ingredients	g/kg as Fed
Corn	626
Gluten meal	20.0
Soybean meal, 48% CP	292
Soya oil	25.0
Di-calcium phosphate	16.5
Limestone	7.00
Salt	4.50
Vitamin–mineral premix *	5.00
L-threonine	0.50
DL-methionine	0.80
L-lysine	1.70
Choline chloride	0.20
3-Phytase	0.80
**Total**	1000
**Nutrient content**	
**Chemical analysis**	
Metabolizable energy (kcal/kg)	3150
Crude protein, g/kg	202
Crude fat, g/kg	58.8
Ash, g/kg	5.63
**Calculated analysis**	
Calcium, g/kg	8.48
Available phosphorus, g/kg	4.21
DL-methionine, g/kg	5.68
L-lysine, g/kg	11.00
Sodium, g/kg	1.40

* Premix provided the following per kg of diet: vitamin A, 1500 IU; vitamin D3, 200 IU; vitamin E, 10 mg; vitamin K3, 0.5 mg; thiamine, 1.8 mg; riboflavin, 3.6 mg; pantothenic acid, 10 mg; folic acid, 0.55 mg; pyridoxine, 3.5 mg; niacin, 35 mg; cobalamin, 0.01 mg; biotin, 0.15 mg; Fe, 80 mg; Cu, 8 mg; Mn, 60 mg; Zn, 40 mg; I, 0.35 mg; Se, 0.15 mg.

**Table 2:** Effect of dietary *Spirulina platensis* supplementation at different levels on growth performance of broiler chickens subjected to cyclic heat stress starting from 22 to 42 days of age.

Traits	TN	HS	HS Added SP, %			NT vs. HS	Effect of SP under Heat Stress	
			0.5%	1%	1.5%	*p*-Value	SEM	*p*-Value
IBW, g	660	640	655	650	644	0.560	96.75	0.124
FBW, g	2325	1860 ^b^	2150 ^ab^	2210 ^a^	2140 ^ab^	0.012	53.25	0.034
ADG, g/d	76.33	54.43 ^c^	61.57 ^b^	69.20 ^a^	62.05 ^b^	0.009	4.86	0.031
FI, g/d	156.58	138.25	139.29	144.45	134.58	0.037	10.49	0.087
FCR	1.87	2.26 ^a^	2.03 ^b^	1.90 ^c^	1.94 ^c^	0.002	0.083	0.023
EBI	394.1	227.3 ^c^	297.2 ^b^	382.7 ^a^	337.2 ^a^	0.007	49.83	0.013

Means within a row with different superscripts significantly differ (*p* < 0.05). SEM, Standard error of the mean, TN, fed the basal diet and reared under thermoneutral condition; HS, cyclic heat stress exposure; SP, *Spirulina platensis*; IBW, initial body weight; FBW, final body weight; ADG, average daily gain; FI, feed intake; FCR, feed conversion ratio; EBI, European broiler index; SEM, Standard error of the mean.

**Table 3:** Effect of dietary *Spirulina platensis* supplementation at different levels on hematological parameters of broiler chickens subjected to cyclic heat stress starting from 22 to 42 days of age.

Parameters	TN	HS	HS Added SP, %			NT vs. HS	Effect of SP under Heat Stress	
			0.5%	1%	1.5%	*p*-Value	SEM	*p*-Value
RBC, 10^6^/mm^3^	2.25	2.14 ^c^	2.35 ^a^	2.27 ^b^	2.25 ^b^	0.047	0.031	0.032
HGB, g/dL	9.7	9.1 ^c^	9.6 ^b^	10.4 ^a^	10.2 ^a^	0.041	0.029	0.033
HCT, %	33.56	28.53 ^b^	31.82 ^a^	32.77 ^a^	30.31 ^a^	0.013	0.232	0.025
MCV, µm^3^/RBC	205.14	191.30 ^b^	215.51 ^a^	207.72 ^a^	200.87 ^b^	0.019	0.563	0.047
MCH, pg	64.2	62.6 ^b^	66.1 ^a^	65.0 ^a^	62.97 ^b^	0.033	0.121	0.036
MCHC, %	32.69	33.30	30.90	31.28	31.83	0.109	0.301	0.110

Means within the same row with different superscripts significantly differ (*p* < 0.05). SEM, Standard error of the mean, TN fed the basal diet and reared under thermoneutral condition; HS, cyclic heat stress exposure; SP, *Spirulina platensis*; RBC, red blood cell; HGB, hemoglobin; HCT, hematocrit; MCV, mean cell volume MCH, mean cell hemoglobin and MCHC: mean cell hemoglobin concentration.

**Table 4:** Effect of dietary *Spirulina platensis* supplementation at different levels on blood serum profile of broiler chickens subjected to cyclic heat stress starting from 22 to 42 days of age.

Parameters	TN	HS	HS Added SP, %			NT vs. HS	Effect of SP under Heat Stress	
			0.5%	1%	1.5%	*p*-Value	SEM	*p*-Value
CHOL, mg/dL	191.3	236.5 ^a^	201.3 ^b^	190.5 ^b^	186.6 ^b^	0.015	4.123	0.022
LDL, mg/dL	91.7	138.6 ^a^	100.2 ^b^	85.2 ^c^	78.5 ^c^	0.011	3.204	0.031
HDL, mg/dL	64.4	61.1 ^c^	65.9 ^b^	70.3 ^a^	73.1 ^a^	0.003	1.378	0.034
TG, mg/dL	176	184 ^a^	176 ^b^	175 ^b^	175 ^b^	0.023	1.123	0.028
AST, U/L	62.4	65.7 ^a^	62.3 ^b^	61.6 ^b^	62.2 ^b^	0.031	0.543	0.045
ALT, U/L	69.70	70.68	71.09	73.97	72.55	0.071	1.137	0.091
Creatinine, mg/dL	0.28	0.46 ^a^	0.28 ^b^	0.25 ^b^	0.27 ^b^	0.002	0.021	0.018
urea, mg/dL	5.35	6.3 ^a^	4.82 ^b^	4.17 ^c^	5.22 ^b^	0.019	2.125	0.023

Means within the same row with different superscripts significantly differ (*p* < 0.05). SEM, Standard error of the mean, TN fed the basal diet and reared under thermoneutral condition; HS, cyclic heat stress exposure; SP, *Spirulina platensis*; CHOL, total cholesterol; LDL, low-density lipoprotein cholesterol; HDL, high-density lipoprotein cholesterol; TG, triglycerides.

**Table 5:** Effect of dietary *Spirulina platensis* supplementation at different levels on blood redox profile of broiler chickens subjected to cyclic heat stress starting from 22 to 42 days of age.

Parameters	TN	HS	HS Added SP, %			NT vs. HS	Effect of SP under Heat Stress	
			0.5%	1%	1.5%	*p*-Value	SEM	*p*-Value
MDA, nmol/mL	1.9	3.5 ^a^	3.1 ^b^	2.7 ^c^	2.8 ^c^	0.021	0.141	0.009
SOD, U/mL	5.2	3.4 ^b^	6.3 ^a^	6.1 ^a^	5.8 ^a^	0.019	0.451	0.004
GSH, µmol/L	37.2	23.3 ^c^	29.7 ^b^	34.6 ^a^	32.1 ^a^	0.029	2.570	0.009
TAC, U/mL	8.4	5.6 ^c^	7.2 ^b^	8.1 ^a^	8.6 ^a^	0.040	0.248	0.011

Means within the same row with different superscripts significantly differ (*p* < 0.05). SEM, Standard error of the mean, TN fed the basal diet and reared under thermoneutral condition; HS, cyclic heat stress exposure; SP, *Spirulina platensis*; MDA, malondialdehyde; SOD, superoxide dismutase; GSH, glutathione reduced; TAC, total antioxidant capacity.

**Table 6:** Effect of dietary *Spirulina platensis* supplementation at different levels on carcass characteristics of broiler chickens subjected to cyclic heat stress starting from 22 to 42 days of age.

Traits (%)	TN	HS	HS Added SP, %			NT vs. HS	Effect of SP under Heat Stress	
			0.5%	1%	1.5%	*p*-Value	SEM	*p*-Value
Dressing	69.01	67.83 ^b^	68.50 ^a^	68.99 ^a^	68.00 ^b^	0.030	0.195	0.020
Breast	36.71	35.63 ^c^	37.94 ^ab^	38.51 ^a^	36.99 ^b^	0.020	0.120	0.032
Leg	30.21	29.31	29.57	29.85	28.38	0.070	0.125	0.091
Fat	2.48	1.95 ^b^	2.45 ^a^	2.39 ^a^	2.40 ^a^	0.011	0.088	0.042
Liver	2.85	2.89	2.90	2.91	2.89	0.083	0.225	0.083
Heart	0.83	0.83	0.84	0.83	0.82	0.231	0.021	0.112
Gizzard	3.71	3.51	3.62	3.72	3.63	0.124	0.141	0.117
Intestine	6.53	6.31 ^b^	7.70 ^a^	7.74 ^a^	7.70 ^a^	0.128	0.148	0.031

Means within the same row with different superscripts significantly differ (*p* < 0.05). SEM, Standard error of the mean, TN fed the basal diet and reared under thermoneutral condition; HS, cyclic heat stress exposure; SP, *Spirulina platensis*.

**Table 7:** Effect of dietary *Spirulina platensis* supplementation at different levels on meat quality of broiler chickens subjected to cyclic heat stress starting from 22 to 42 days of age.

Traits	TN	HS	HS Added SP, %			NT vs. HS	Effect of SP under Heat Stress	
			0.5%	1%	1.5%	*p*-Value	SEM	*p*-Value
pH 24 h	5.78	5.76	5.80	5.79	5.74	0.070	0.161	0.119
Lightness (L^*^)	49.3	48.5	47.4	46.8	47.1	0.170	1.318	0.074
Yellowness (b^*^)	4.37	4.40 ^c^	10.68 ^b^	11.59 ^a^	12.24 ^a^	0.290	0.228	0.031
Redness (a^*^)	4.52	4.49	5.32	5.48	5.36	0.120	0.509	0.101
Cooking loss,%	14.6	12.5	13.5	12.3	12.6	0.100	0.657	0.121
Shear force, kg	1.64	1.59	1.61	1.55	1.58	0.140	0.217	0.108

Means within the same row with different superscripts significantly differ (*p* < 0.05). SEM, Standard error of the mean, TN fed the basal diet and reared under thermoneutral condition; HS, cyclic heat stress exposure; SP, *Spirulina platensis*.

## Data Availability

All data sets obtained and evaluated during the current study are available upon appropriate request from the corresponding author. All data sets obtained and evaluated during the current study are available upon appropriate request from the corresponding author.
